# Intraoperative Blood Loss during Induced Abortion: A Comparison of Anesthetics

**DOI:** 10.1155/2018/4051896

**Published:** 2018-12-02

**Authors:** Camille A. Clare, Gabrielle E. Hatton, Neela Shrestha, Michael Girshin, Andre Broumas, Danielle Carmel, Mario A. Inchiosa

**Affiliations:** ^1^Department of Obstetrics and Gynecology, New York Medical College, Valhalla, New York, USA; ^2^Department of Surgery, The University of Texas at Houston/UTHealth, Houston, Texas, USA; ^3^Department of Anesthesiology, New York Medical College, Valhalla, New York, USA; ^4^Department of Pharmacology, New York Medical College, Valhalla, New York, USA

## Abstract

**Objective:**

To determine whether there is a difference in intraoperative bleeding with inhalational versus noninhalational anesthetic agents for patients undergoing suction dilatation and curettage for first-trimester induced abortion.

**Methods:**

This is an IRB-approved retrospective chart review of the electronic medical records of patients undergoing induced abortion at gestational ages between 5 0/7 and 14 0/7 weeks of pregnancy at the New York City Health + Hospitals/Metropolitan. The records of 138 patients who underwent suction dilatation and curettage for induced abortion between June 2012 and June 2014 were reviewed for an association between anesthetic technique and intraoperative hemorrhage. Twenty patients received inhalational anesthetic agents, while 118 received intravenous anesthetics. Blood loss was estimated by the operating gynecologists.

**Results:**

The mean intraoperative blood loss for inhalational anesthetics (113.6 ml) was significantly higher than with noninhalational agents (40.2 ml) (*p*=0.007). Age, body mass index, and gestational age were not statistically different between the groups; the number of methylergonovine doses at induced abortion trended higher with inhalation anesthetics.

**Conclusions:**

The difference in blood loss between the two types of anesthetic techniques was statistically significant. These findings may be important for patients with significant anemia or at an increased risk of bleeding, such as those with unrecognized coagulopathies.

## 1. Introduction

Over one million pregnancies result in induced abortion in the United States each year [1]. Recent studies have shown that the mortality risk from an induced abortion is 0.7 per 100,000 [2]. Minor complications have dropped to an estimated eight per 1000 abortions by 1990, and major complications have decreased to 0.7 per 1000 induced abortions [3]. Both hemorrhage and anesthetic complications have been the leading causes of abortion-related mortality. Hemorrhage is the leading cause of mortality during second-trimester induced abortions, and is the second leading cause of mortality in first-trimester induced abortions, following infection [4]. Recent studies have documented the continued importance of postabortion hemorrhage; a conservative estimate of the incidence of hemorrhage of clinical consideration is approximately 1% of procedures [5, 6]. A large study reported the incidence of hemorrhage requiring blood transfusions at 0.4% [7].

Many studies have compared the effects of volatile and intravenous anesthetic agents on isolated uterine myometrium in vitro [8–10]. The use of volatile agents, such as halothane and enflurane, has led to significantly increased blood loss. All inhalational agents were used at 1.5 MAC and above, and such concentrations would likely be avoided in current practice. The following anesthesia concentrations were noted in a Hall et al. study: patients received fentanyl one microgram/kilogram (kg), followed by an induction dose of propofol (2-3 mg/kg); then a propofol infusion was started using a standard regimen of 12 mg/kg/h for 10 min and then 9 mg/kg/h for 10 min; they also received bolus doses of propofol (20 mg) if anesthesia was too light; patients breathed 66% nitrous oxide in oxygen via face mask; in the second group, after preoxygenation, patients received fentanyl one microgram/kilogram followed by an induction dose of propofol (2-3 mg/kg); anesthesia was maintained with 1% isoflurane (measured concentration) and 66% nitrous oxide in oxygen via face mask; isoflurane concentration was increased by 0.5% and a further 0.5% if necessary, using a concentration of 1% isoflurane if the anesthesia was too light and when the patient was stable [11]. Few international studies have compared volatile anesthetics with propofol for minor gynecologic procedures, including induced abortion [12]. One Canadian study by Nathan et al. was prematurely terminated in a prospective randomized study comparing the cost of sevoflurane and propofol during ambulatory induced abortion procedures due to an unacceptable increase in bleeding in the sevoflurane group [13]. Another prospective study completed in Wales by Hall et al. found significantly more intraoperative blood loss with the use of inhaled isoflurane than with intravenous propofol [11]. The existing literature is approximately 20–30 years old, and the dosages cited are no longer recommended. The current study describes the up-to-date use of these anesthetic agents for this important patient group.

Also, one of the authors (MAI) was a coauthor in a major recent study that examined the frequency of unrecognized abnormal coagulation indices in patients who had been prescreened and deemed negative by their history and physical findings for coagulation defects [14]. A relatively high incidence of unanticipated abnormal coagulation indices was observed and prompted the authors to report findings on the possible adverse influence of the anesthetic intervention on some members of a patient population that may be more vulnerable to excessive bleeding, such as during an induced abortion.

## 2. Materials and Methods

Following Institutional Review Board (IRB) approval (L-11,256) from New York Medical College, New York City Health + Hospitals/Metropolitan, and the New York City Health + Hospitals, a single institution retrospective chart review was conducted. Patients who had complete anesthetic records and underwent suction dilatation and curettage for induced abortion from 5 0/7 weeks to 14 0/7 weeks of gestation between June 1, 2012, and June 1, 2014, were evaluated. A waiver of informed consent was obtained per IRB guidelines due to the retrospective chart review.

Sedation state and/or general anesthesia was maintained with intravenous (IV) propofol, inhaled sevoflurane, or inhaled desflurane. Inhaled sevoflurane and desflurane were grouped together for data analysis under inhalational agents. The type of anesthetic agent used was based on the preference and judgment of the attending anesthesiologists. The Department of Anesthesiology for New York City Health + Hospitals/Metropolitan clinical guidelines recommend the use of inhalational agents for induced abortions and dilatation and curettage procedures at equal to or less than 0.75 MAC of the inhalational agent of choice. Complete anesthetic records were available for review, including the documentation of the types of anesthetics used and estimated blood loss. Subjects were excluded from review if there was a lack of hospital records for both anesthetic and blood loss documentation.

The following factors were recorded and analyzed in relation to anesthetic choice and intraoperative blood loss: age, weight, gravidity, parity, past medical history (with an emphasis on a history of bleeding disorders) and past surgical history, gestational age determined via first-trimester ultrasound, preoperative medications, intraoperative medications, postoperative medications, and the American Society of Anesthesiologists (ASA) status. The two coauthors (CAC and AB), who were the operating surgeons, and research assistants (GH and DC), performed the chart review. Data were transferred on a piloted form on an electronic database by the secondary author (GH). The accuracy of the original data transfer and recording was confirmed by the primary author (CAC).

The intraoperative blood loss was estimated by two attending gynecologists (AB and CAC), who performed these procedures. They made a visual estimation of blood collected in a vacuum canister, which was marked off in milliliters and accurately recorded. Their estimations were systematically similar to each other in the assessment of blood loss. The gynecologic surgeons did not measure the postprocedure blood loss. For first-trimester induced abortions, the vacuum canister includes amniotic fluid and products of conception, which is in small amounts when compared to procedures at later gestational ages. The mean gestational ages were the same in the two treatment groups, which did not change the outcome of the estimated intraoperative blood loss significantly.

Patients varied in their past medical and surgical histories. Preoperative complete blood counts (CBC) were obtained in most patients with only two patients having a preoperative hemoglobin level of less than 10 grams per deciliter. Each patient was given an oral azithromycin two gram dose from zero to 24 hours prior to procedure and a prescription for oral methylergonovine 0.2 mg every six hours for four days following procedure. Midazolam and fentanyl were used in most subjects in conjunction with the anesthetic agent of choice.

Statistical analyses were carried out using StatMost © software (Dataxion Corp., Los Angeles, California). A comparison of blood loss in relation to anesthetic technique was done using Student's *t*-test since the data in both groups were normally distributed. The possible contribution of differences in the age of the patient, body mass index (BMI), or gestational age were analyzed with the nonparametric Mann–Whitney test since some of the data were not normally distributed. The need for the use of methylergonovine in the operating room or in the postanesthesia care unit as a possible confounder was analyzed with the Fisher's exact test.

A two-tailed *p* value of 0.05 or less was taken as a measure of statistical confidence for all analyses.

## 3. Results

A total of 197 patients had first-trimester induced abortions in the time frame of the study. Of these, 182 patients received a prescription for methylergonovine postoperatively on discharge from the hospital. One hundred thirty-eight subjects, with electronic medical records and complete anesthesia records and estimated blood loss, were analyzed. Subjects overall ranged from 15 to 43 years; gestational ages ranged from 41 to 89 days; and BMI indexes ranged from 17 to 48 kg/m^2^. The two groups of patients showed no statistical differences in regard to these three parameters (Table 1).

**Figure 1:**
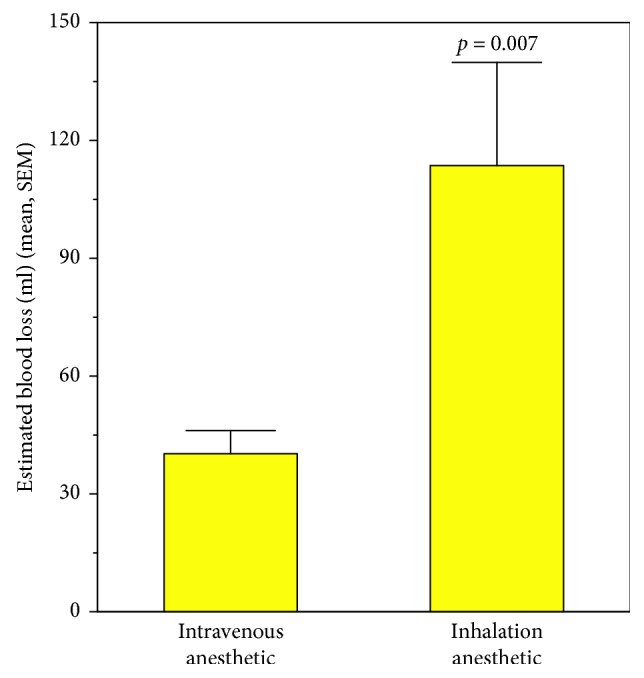
Comparison of blood loss between anesthetics.

Estimated intraoperative blood loss ranged from 5 to 350 ml. The mean estimated blood loss for the inhalational anesthetics was 113.6 ml, while the mean estimated blood loss for the use of intravenous anesthetics was 40.2 ml. This difference was statistically significant (*p* < 0.007; Figure 1).

**Figure 2:**
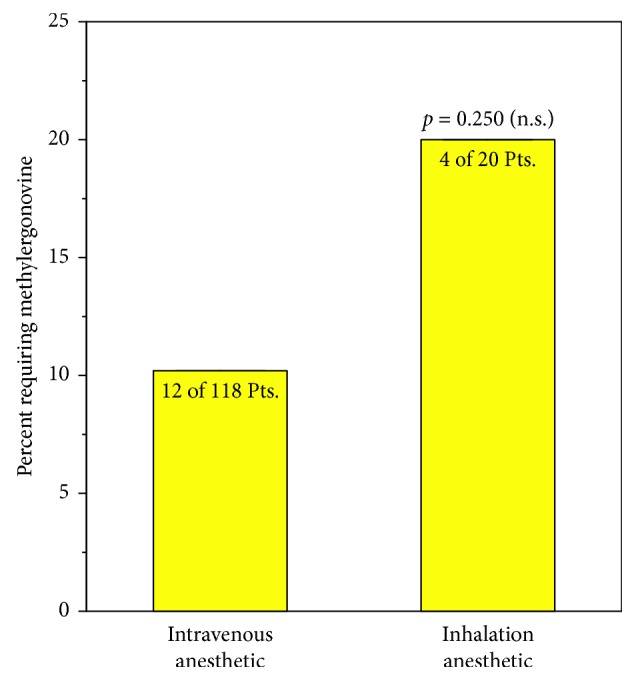
Comparison of perioperative methylergonovine doses.

Twenty percent (4 of 20) of the group receiving inhalation anesthetics received intraoperative doses of methylergonovine compared to 10.2 percent (12 of 118) in the IV anesthetic group (not statistically different; *p*=0.250) (Figure 2).

Nine patients had a suction dilation and curettage at a gestational age of greater than 77 days and received Laminaria japonicum (Cooper Surgical, Inc, Trumbull, CT 06611, USA) for mechanical cervical dilatation at approximately 24 hours before the procedure. Postoperative complete blood counts (CBCs) were not done. None of the patients demonstrated symptoms of acute blood loss anemia or required transfusion.

## 4. Discussion

Our study found a statistically significant difference in the blood loss in patients receiving inhalational versus noninhalational agents. Although total blood loss in both groups was not excessive, the greater blood loss with inhalation anesthetics may be of importance when approaching the patient at an increased risk of uterine bleeding during gynecologic procedures, such as those with recognized or unrecognized coagulopathies. Although increasingly rare, uterine hemorrhage is the leading cause of mortality during second-trimester induced abortions, and is the second leading cause of mortality in first trimester induced abortions.

The risk of unrecognized coagulation issues was documented in a multicenter study involving more than one million patients that included data on the incidence of unanticipated abnormal prothrombin (PT) or activated partial thromboplastin time (aPTT) coagulation indices in presurgical testing [14]. As noted above, our institution was a participant in that study. In patients that did not have history and physical findings or laboratory results that would prompt either a PT test (i.e., history of abnormal bleeding, warfarin therapy, clotting factor deficiencies, and liver disease) or aPTT test (i.e., on heparin therapy, hemophilia, evidence of lupus anticoagulant factor, or von Willebrand disease), 6.6% of the patients had abnormal PT values and 7.1% had abnormal aPTTs [14].

Optimizing anesthetic choice and addressing hemorrhage risk factors prior to a procedure may reduce the incidence of hemorrhage-related morbidity and mortality. Our results suggest that patients with anemia or with increased risk of bleeding should not be given inhalational anesthesia unless other factors outweigh this increased risk of bleeding. Other intraoperative options to address bleeding are the use of intracervical vasopressin or intravenous oxytocin (although the latter depends on the gestational age, the presence of oxytocin receptors, and the timing of the pregnancies) in patients thought to be at increased risk of bleeding during the induced abortions. The current study lends emphasis to the danger that patients with compromised hemostasis might face if treated with inhalational anesthesia. It is one more considerable factor for the anesthesiologist when making individualized decisions on which anesthetic technique to utilize.

In vitro studies may point to a mechanism behind these results. A study by Paull and Ziccone revealed the depressant effects of volatile anesthetics on isolated human uterine muscle contractility [15]. It has been hypothesized that inhalational anesthetics result in the relaxation of uterine muscle and predispose the gravid uterus to hemorrhage. Micks et al. also demonstrated that the use of sevoflurane slightly resulted in a measured blood loss of over 300 ml (considered to be excessive) or increased the frequency of interventions to treat hemorrhage in patients who underwent induced abortions at 18 to 24 weeks of gestation, although this was not statistically significant [16]. This study was also underpowered to detect these differences. Also, at higher gestational ages, such as when induced abortions were performed in the Micks et al. study, there is a higher risk of bleeding and excessive blood loss [16]. The differences in blood loss between the inhalational agents and noninhalational agents were still noted in patients who received more doses of methylergonovine in our study since halogenated inhalation anesthetics exert a direct relaxation of the myometrium [8–10]. This opposes, in a functional (physiological) manner, the myometrial contractile efficacy expected from methylergonovine and, thus, leads to more blood loss.

One limitation of this study was that the gestational ages of patients who underwent induced abortions were at or below 14 and 0/7 weeks. Atony has been associated with an increased risk of hemorrhage in a review of 3000 surgical abortions in the second trimester in patients that were of older maternal age and greater gestational ages [17]. Another limitation was the measurement of blood loss, which although carefully estimated by two attending gynecologists performing the procedures, is known to have inherent problems with accuracy. Another limitation of our study was that the gynecologic surgeons were not blinded for the choice of anesthetic provided.

In view of the relatively small number of patients represented in the group receiving inhalation anesthetics and the lack of statistically significant differences between the patient groups for factors that might be expected to influence blood loss (i.e., age, BMI and gestational age; Table 1), we conducted power analyses on these three comparisons to evaluate the possible risk of Type II errors. Assuming the same variances in the comparisons and preserving the same ratio between the numbers in the two groups, it would require a total sample of 3,414 patients to bring the small difference between the mean values for age to statistical significance. Likewise, 3,567 patients would be required for a statistical difference between the groups for BMI, and 9,540 patients required for the difference in gestational age. These analyses are supportive that our findings are not diminished by Type II errors. And, as noted above, the finding of a statistically significant difference in blood loss between the two groups (Figure 1) is strengthened by the fact that the data in both groups were normally distributed and not influenced by several extreme cases of blood loss.

The group that received inhalation anesthetics had greater blood loss despite the fact that they had approximately twice the frequency of use of doses of methylergonovine than the group receiving IV anesthetics (Figure 2). This difference in frequency did not reach statistical significance; however, as part of our power analyses, we found that if the total patient number (with the same variances and ratio of IV to inhalation anesthetic) was three times as large as in the present data, the trend toward higher use of methylergonovine would reach a statistical difference.

In summary, choice of the anesthetic should be considered in all gynecologic procedures, particularly for induced abortions at both early and late gestational ages, to reduce the potential risk of morbidity and mortality. Our findings extend the advisability of avoidance of inhalational anesthetics for induction of abortion where possible. Anesthesiologists, in particular, should consider the increased risk of bleeding associated with inhalation anesthesia over noninhalational agents when individualizing the anesthetic technique for patients presenting for induced abortion, especially those with anemia or increased risk of bleeding. This is emphasized by the relatively high frequency of abnormal coagulation indices that were found in the large multicenter study noted above in patients who were cleared on the basis of standard criteria that would prompt PT or aPTT testing.

## Figures and Tables

**Table 1:** Absence of differences in demographic factors between the anesthetic treatments.

	Intravenous anesthetic, *n*=118	Inhalation anesthetic, *n*=20	Group comparisons
	Mean (SE)	Mean (SE)	*p* value
Age (year)	27.8 (0.6)	28.6 (1.4)	0.444
BMI (kg/m^2^)	27.0 (0.6)	26.2 (1.1)	0.749
Gestational age (days)	58.6 (1.3)	57.4 (2.8)	0.470

## Data Availability

The data used to support the findings of this study are available from the corresponding author upon request.
